# Kermit Interacts with Gαo, Vang, and Motor Proteins in *Drosophila* Planar Cell Polarity

**DOI:** 10.1371/journal.pone.0076885

**Published:** 2013-10-03

**Authors:** Chen Lin, Vladimir L. Katanaev

**Affiliations:** 1 Department of Pharmacology and Toxicology, Faculty of Biology and Medicine, University of Lausanne, Lausanne, Switzerland; 2 Institute of Protein Research, Russian Academy of Science, Pushchino, Russia; University of Bern, Switzerland

## Abstract

In addition to the ubiquitous apical-basal polarity, epithelial cells are often polarized within the plane of the tissue – the phenomenon known as planar cell polarity (PCP). In *Drosophila*, manifestations of PCP are visible in the eye, wing, and cuticle. Several components of the PCP signaling have been characterized in flies and vertebrates, including the heterotrimeric Go protein. However, Go signaling partners in PCP remain largely unknown. Using a genetic screen we uncover Kermit, previously implicated in G protein and PCP signaling, as a novel binding partner of Go. Through pull-down and genetic interaction studies, we find that Kermit interacts with Go and another PCP component Vang, known to undergo intracellular relocalization during PCP establishment. We further demonstrate that the activity of Kermit in PCP differentially relies on the motor proteins: the microtubule-based dynein and kinesin motors and the actin-based myosin VI. Our results place Kermit as a potential transducer of Go, linking Vang with motor proteins for its delivery to dedicated cellular compartments during PCP establishment.

## Introduction

Cell polarization is essential for tissue development and function. Apart from the ubiquitous apical-basal polarity, epithelial cells can also polarize within the plane of the epithelium. This phenomenon is known as planar cell polarity (PCP) or tissue polarity and was first identified in *Drosophila* where mutations in PCP genes affect the uniform arrangement of ommatidia in eyes, anterior-posterior organization of sensory cuticle bristles, and proximo-distal orientation of wing hairs [1,2]. PCP signaling regulates several developmental processes also in vertebrates, such as convergent extension during gastrulation [3] and organization of sensory cells in the inner ear [4]. Extensive studies in *Drosophila* have uncovered several core PCP components: the transmembrane proteins Frizzled (Fz), Van Gogh (Vang, also known as Strabismus), and Flamingo; and cytosolic proteins Dishevelled, Prickle, and Diego [5]. A number of other proteins have been implicated in PCP signaling, such as Go – the heterotrimeric G protein serving as an immediate transducer of Fz [6,7], actin cytoskeleton regulator RhoA [8], and small GTPases Rab5 and Rab11 regulating vesicular trafficking during PCP establishment [9]. The latter appears particularly important, as several PCP transducers have been found to relocalize to specific sites during PCP establishment from their initial distributions [10] – the process required to amplify the initial cell’s polarization and relying on the cytoskeleton and the cytoskeleton-based motor proteins [11]. These redistributions are exemplified by the distal accumulation of Fz and proximal – of Vang [12,13].

Fz and other proteins of this family are atypical G protein-coupled receptors (GPCR) [14]. Fz proteins bind heterotrimeric G proteins and activate them [15-17]. In *Drosophila*, the heterotrimeric Go protein was implicated in Fz signal transduction [6,7,9,18-20] – the finding corroborated by similar observations in other organisms [21-25]. A heterotrimeric G protein consists of a guanine nucleotide-binding α-subunit and the βγ-heterodimer. Upon activation with a GPCR, GDP on the α-subunit becomes exchanged with GTP, leading to dissociation of Gα-GTP and Gβγ into free signaling-competent transducers. Gα-GTP is deactivated through GTP hydrolysis – reaction catalyzed by the RGS (Regulator of G-protein Signaling) proteins [26].

To identify potential signaling partners of Go in *Drosophila* PCP, we performed a genetic screen with overexpression of the α-subunit of Go (Gαo) and uncovered Kermit as a new interaction partner. Kermit is the *Drosophila* homolog of GIPC – a mammalian PDZ domain-containing protein first discovered to interact with GAIP/RGS19, one of the RGS family members acting on several G proteins including Gαo [27]. Subsequent studies revealed that Kermit/GIPC could also interact with Fz3 and Fz7 in *Xenopus* [28]. As both Kermit and RGS19 were implicated in PCP signaling [29,30], complex interactions involving Fz, Gαo, GAIP/RGS19, and Kermit/GIPC could be anticipated to mediate PCP signaling. However, *kermit* loss-of-function mutants in *Drosophila* are viable without any obvious phenotypes [29], suggesting that Kermit may play a redundant regulatory function in PCP. Here we analyze the Kermit/Gαo interaction in *Drosophila* PCP and provide evidence for the role of Kermit in motor protein-based relocalization of Vang.

## Results

### Identification of kermit as a suppressor of *Gαo* phenotypes

**Figure 1:**
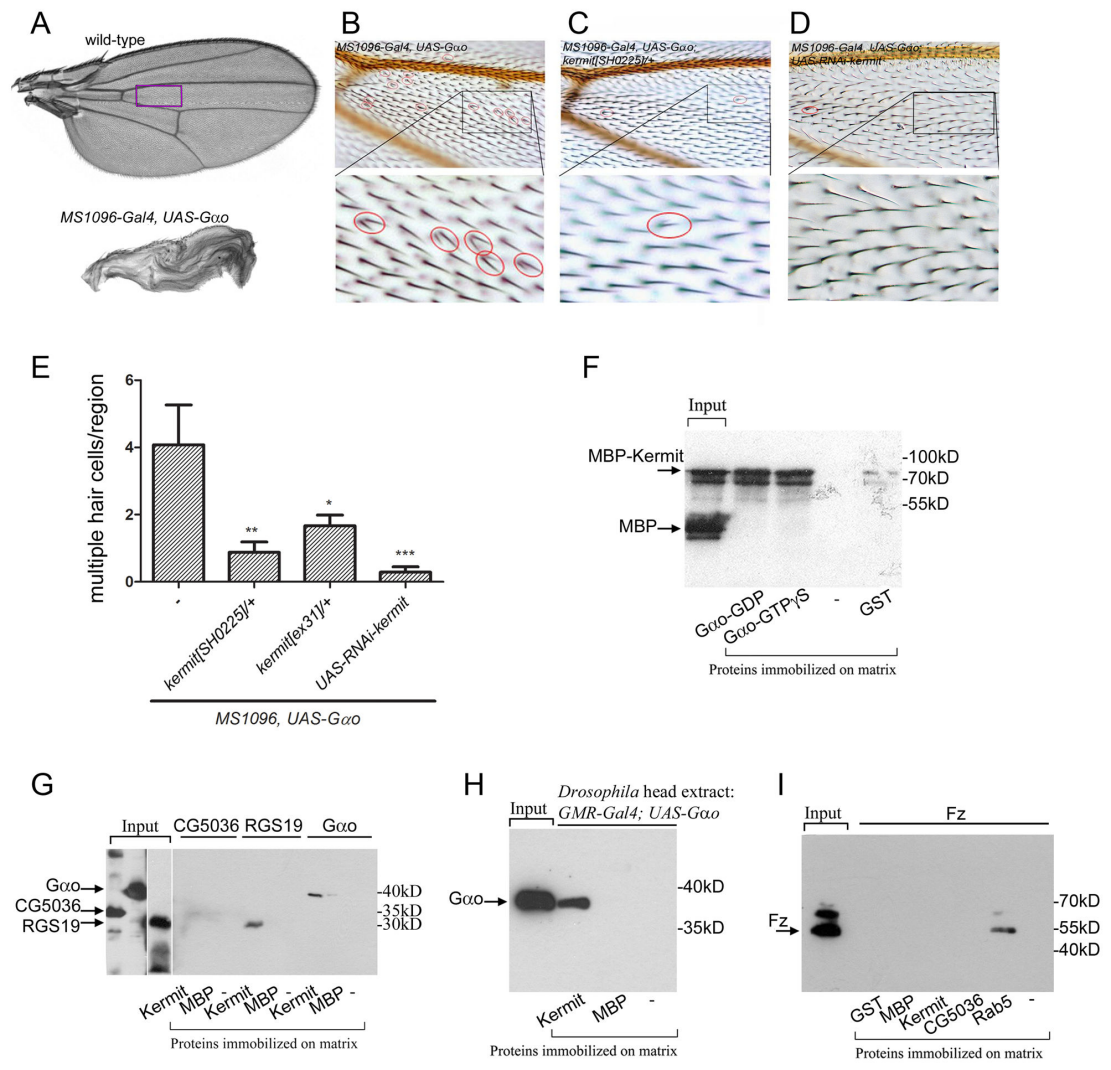
Kermit is identified as a novel binding partner of Gαo. Overexpression of *Gαo* in *Drosophila* wings leads to folded wings (A) and multiple hair cells (B). The magenta frame on the wild-type wing in (A) indicates the region magnified in (B-D). The multiple hair phenotype is strongly suppressed in a *kermit* heterozygous mutant (C) or RNAi against *kermit* (D) background. Lower panels in (B, C, D) show higher magnification of the selected regions of the wings. (E) Quantification of the multiple hair cells induced by *Gαo* overexpression by the *MS1096-Gal4* driver in different genotypes. Statistical significance was assessed by the Student’s t-test; “***” indicates P-value < 0.0005; “**” indicates P-value ˂ 0.005; “*” indicates P-value ˂ 0.05. (F) Expression/purification of MBP-Kermit produces a mixture of the fusion protein and cleaved MBP (arrows), the latter serving as an internal binding control. In pull-down assays, MBP-fused recombinant Kermit, but not MBP itself, indiscriminately binds to GDP- or GTPγS-loaded Gαo-matrices, but not to control GST-loaded or empty matrices. (G) Immobilized Kermit was able to pull down soluble Gαo and human RGS19, but not CG5036. (H) Kermit and control proteins were immobilized on matrix to pull-down Gαo from head extracts of *Drosophila* overexpressing *Gαo* in the eyes (using the *GMR-Gal4* driver). (I) Solubilized Fz failed to be precipitated by Kermit or CG5036, but was bound by Rab5.

Overexpression of *Gαo* in *Drosophila* wings leads to a folded-wing phenotype, when flies fail to expand their wings after emergence from the pupal case [31] (Figure 1A). We used a collection of 619 mutations of essential genes from the Szeged stock center, estimated to cover ca. 50% of the second chromosome essential genes and ca. 25% of the total vital genes of the *Drosophila* genome [32], to screen for mutations which when heterozygous would suppress the folded-wing phenotype of *Gαo* overexpression. Detailed results and analysis of this screen will be published elsewhere. One of the mutations found to suppress the folded-wing phenotype was *kermit*[*SH0225*]. While 78% of the control adult *MS1096-Gal4, UAS-Gαo/X* flies had folded wings, this number decreased to 22% in the *MS1096-Gal4, UAS-Gαo/X; kermit*[*SH0225*]*/+* flies. Independent repetition of the cross confirmed this finding; statistical analysis revealed that the result is highly significant (P value <0.0001 by the Pearson’s chi-squared test).

Overexpression of *Gαo* also leads to a dominant PCP phenotype, seen as formation of multiple hairs by individual wing cells [6]. This phenotype can be interpreted as the cell’s inability to form a single polarization focus [7,18]. This multiple hair phenotype is suppressed several-fold by *kermit*[*SH0225*] (Figure 1B, C, E), as well as by another allele *kermit*[*ex 31*] (Figure 1E) in the heterozygous *kermit* mutant backgrounds. Further, co-expression of an RNAi targeting *kermit* leads to a near-complete suppression of the multiple wing hair phenotype (Figure 1D, E).

Kermit is an evolutionary conserved PDZ domain-containing protein implicated in numerous protein-protein interactions [33]. In *Drosophila*, *kermit* is strongly upregulated in the developing wing between 24 and 40h after puparium formation – the time of PCP establishment [34]. GIPC, the mammalian homolog of Kermit, binds to RGS19 which is in turn implicated in mammalian Fz signaling [27,30]. Further, GIPC/Kermit could also interact with *Xenopus* Fz3, Fz7, and other GPCRs [28,33]. Thus, a model could be formulated that a quaternary complex among Fz, Gαo, GAIP/RGS19, and Kermit/GIPC could form and mediate PCP signaling.

### Kermit physically interacts with Gαo but not Fz or CG5036

To test the validity of this model, we performed a series of *in vitro* interaction experiments between Kermit and its potential binding partners. First, to check whether the genetic interaction between *Gαo* and *kermit* is paralleled by their physical binding, we purified Kermit as an MBP (maltose-binding protein)-tagged recombinant protein after bacterial expression. Recombinant Gαo was also purified and immobilized on CNBr-Sepharose – procedure leading to active Gαo competent to interact with guanine nucleotides and partner proteins [20]. We found that MBP-Kermit, but not MBP itself, bound equally to the GDP- or GTP-loaded forms of Gαo but not control (empty or GST-loaded) matrices (Figure 1F). Similar interaction was observed when Kermit was immobilized on amylose resin to pull-down Gαo (Figure 1G). Additionally, recombinant Kermit could pull-down endogenous Gαo from *Drosophila* head extracts (Figure 1H).

To determine whether GIPC/Kermit interaction with RGS19 is conserved in *Drosophila*, we cloned and purified RGS19 as well as its *Drosophila* homolog CG5036. However, CG5036 lacks the atypical PDZ-binding motif of RGS19 at the C-terminus (Ser-Glu-Ala in RGS19 *vs.* Ser-Pro-Thr in CG5036), as well as any typical PDZ-binding motifs [35]. Concordantly, we failed to detect any interaction of CG5036 with Kermit, although RGS19 demonstrated such interaction (Figure 1G). Further, we failed to detect a physical interaction between *Drosophila* Kermit and Fz, although Fz revealed robust interaction with Rab5 in these conditions as previously reported [9] (Figure 1I). Thus, both the Fz-Kermit and the RGS-Kermit vertebrate interactions are not conserved in *Drosophila*.

### Upregulation of kermit produces dominant PCP phenotypes enhanced by overexpression of *Gαo*

**Figure 2:**
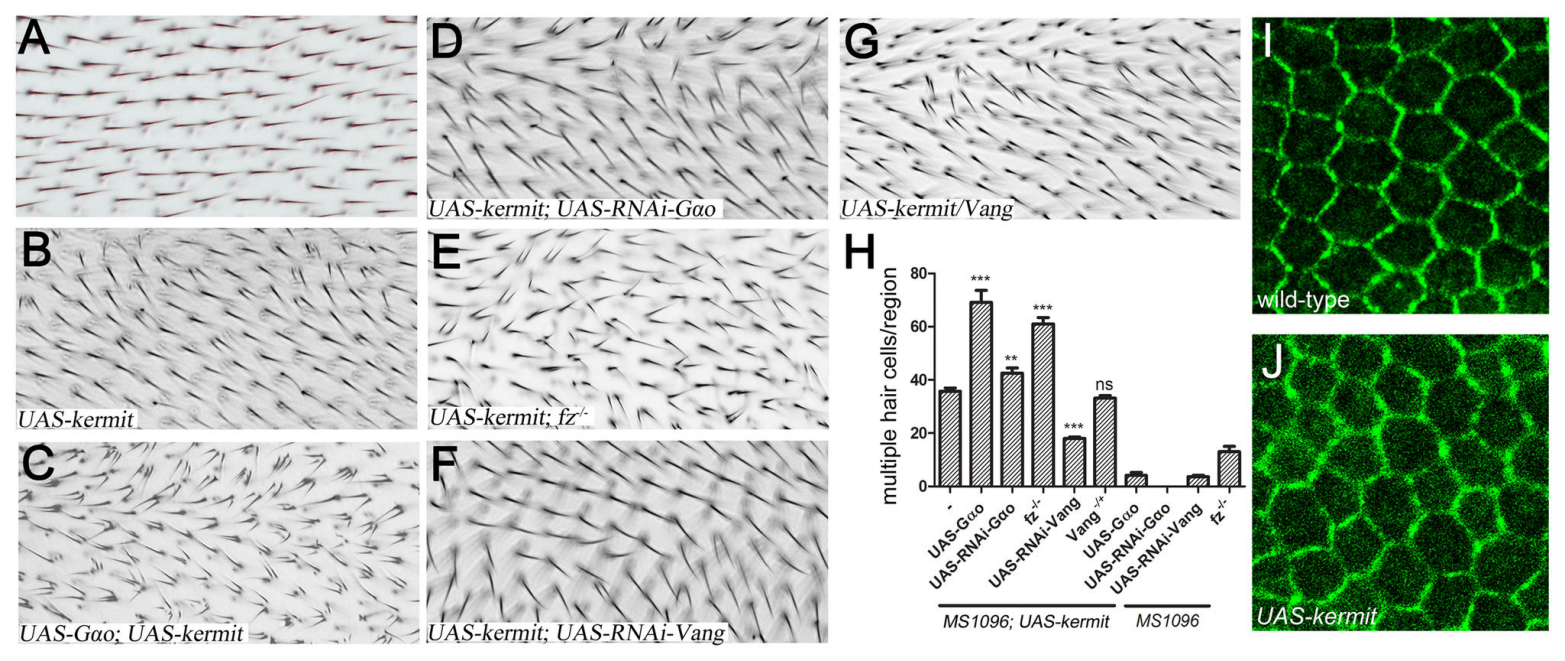
Kermit appears to act downstream from Gαo but upstream from Vang. Wild-type (*yw*) wing hairs display uniform proximal to distal orientation (A). Overexpression of *kermit* under the *MS1096-Gal4* driver control results in strong PCP phenotypes including swirling and cells with multiple hairs (B). The *UAS-kermit* phenotypes are dramatically enhanced by co-overexpression of *Gαo* (C), but not by its downregulation (D). These phenotypes are also enhanced upon removal of *fz* (E). Reduction of *Vang* suppresses the phenotype if achieved by RNAi-mediated downregulation (F), but not by a mere removal of one gene copy (G). The panels (A-G) represent high-magnification images of the dorsal wing sheet within the region framed in magenta in Figure 1A. (H) Quantification of the multiple hair cells induced by Kermit in different genotypes, presented as on Figure 1E. (I, J) Vang-YFP localization in pupal wings of the wild-type (I) and *UAS-kermit* genotypes (J). Distal is right, anterior is up.

An enhancer-trap line of *kermit* was reported to induce PCP defects seen as hair swirling and multiple wing hair formation, while a direct *UAS-kermit* transgene induced less pronounced PCP defects [29,36]. Intriguingly, *kermit* loss-of-function alleles *kermit*[*ex 2*] and *kermit*[*ex 31*] were homozygous viable without any discernible phenotypes [29], contrasting the lethality of the *kermit*[*SH0225*] allele [32] and hinting at potential redundancy in the PCP signaling. To understand the role of Kermit *in vivo*, we generated our own transgenic *UAS-kermit* flies. Overexpression of *kermit* by the wing *MS1096-Gal4* driver produced strong PCP phenotypes including swirling and multiple hairs (Figure 2B). As expected from the *kermit*/*Gαo* interactions described above, co-overexpression of the two proteins further aggravated the phenotypes, significantly increasing the number of cells producing multiple hairs (Figure 2C, H). Also, severe re-orientation of hairs was seen in some wing regions (Figure 2C). Moreover, the *UAS-Gαo; UAS-kermit* cells were often forced to produce three hairs (on average, 2.7±0.1), while *UAS-kermit* alone typically induces two (Figure 2B, C; on average 2.2±0.1; the difference being highly statistically significant, with the P value <0.0005 form the Student’s t-test). In contrast, downregulation of *Gαo* using an RNAi construct (whose efficiency has been tested previously [9]) failed to markedly affect the *UAS-kermit* phenotype (Figure 2D, H). Together with the data that the *UAS-Gαo* phenotypes are suppressed upon *kermit* downregulation (Figure 1A-E), these results suggest that *Gαo* acts upstream but not downstream from Kermit in PCP.

We next tested whether a genetic interaction between *kermit* and *fz* existed, despite the lack of a physical Kermit/Fz interaction (Figure 1I) and the reported lack of their genetic interaction [29]. We argued that if Kermit is a simple transducer of Fz in PCP, the *UAS-kermit* phenotype should remain the same upon removal of Fz. If, on the other hand, Kermit is involved in Fz relocalization during PCP, removal of Fz should suppress/abrogate the *UAS-kermit* phenotype. Remarkably, we found a third outcome – that elimination of the Fz protein enhanced the *UAS-kermit* phenotype (Figure 2E, H). Although *fz* ^*-/-*^ wings themselves produce a certain amount of cells with multiple hairs [37], the effect is more than additive (Figure 2H). We tend to interpret this observation as follows: Kermit is not involved in Fz relocalization, but may control the activity of another PCP component antagonized by Fz. Such antagonism between PCP components localizing distally (like Fz) and those localizing proximally during PCP establishment has been previously demonstrated [38,39]. Since Vang epitomizes the proximal localization [13], we set to investigate a possible genetic link between this protein and Kermit.

### Vang likely acts downstream from Kermit

Interaction between mammalian homologs of Kermit and Vang (GIPC1 and Vangl2, respectively) has been recently shown to control Vangl2 trafficking in the inner ear [40]. In *Drosophila* wing epithelia during PCP establishment, Vang is relocalized to the proximal site of cells [13], opposite to Fz localization, by a mechanism which is still unclear. In mammals, the GIPC1-Vangl2 interaction is mediated by the PDZ-binding C-terminal motif of Vangl2 (Glu-Thr-Ser-Val) [35,40], which is conserved in *Drosophila* Vang. We hypothesize that in *Drosophila*, Kermit may similarly regulate Vang trafficking. In this case, the dominant PCP phenotypes of *kermit* overexpression should be diminished upon reduction in Vang levels. Indeed, we found that *Vang* downregulation by RNAi led to a ca. two-fold reduction in multiple hair cells induced by *UAS-kermit* (Figure 2F, H). The *UAS-RNAi-Vang* construct we used was potent on its own to induce PCP defects such as multiple hairs (Figure 2H) and hair disorientation (not shown). In contrast, removal of one gene copy of *Vang* did not affect the *UAS-kermit* phenotype (Figure 2G, H), agreeing with the previous observation [29]. Thus a significant reduction in Vang levels is required to reveal the dependency of the *kermit* overexpression phenotypes on *Vang*. These results suggest that Vang may act downstream of Kermit, and that Kermit may regulate Vang trafficking in *Drosophila* similarly as in the mammalian inner ear. In agreement with these genetic interactions, we find that *kermit* overexpression indeed affects Vang localization: in pupal wings 30h after puparium formation, Vang changes its stereotypical localization at the proximal apical membrane (appearing as the zigzag staining orthogonal to the proximo-distal axis [13] (Figure 2I)) to more diffuse and more random localization (Figure 2J).

### Motor proteins differently affect Kermit activity in PCP

**Figure 3:**
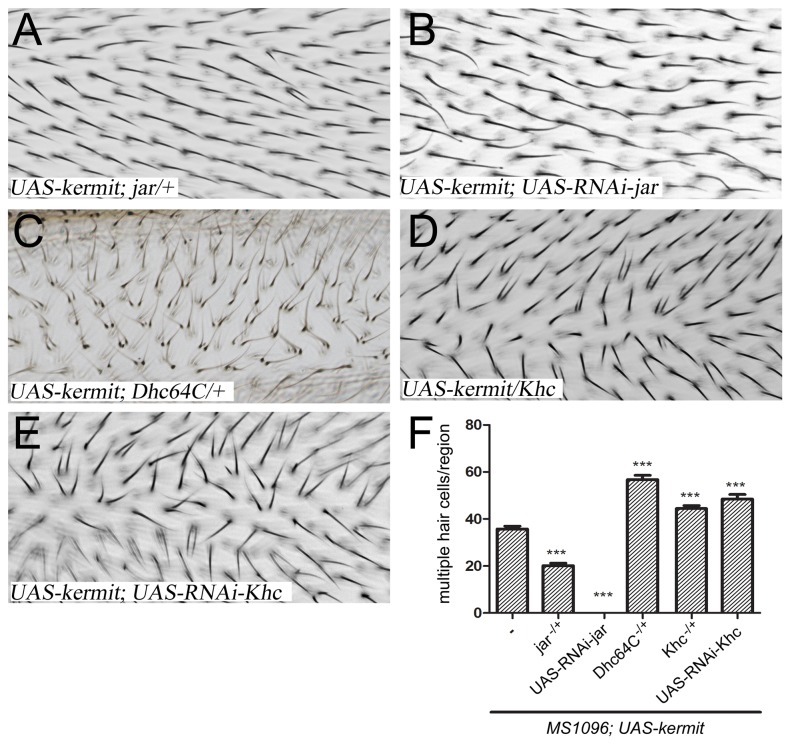
Kermit activity is differently affected by actin- and microtubule-based motors. Downregulation of *jar* (MyoVI) by removal of one gene copy (A) or RNAi (B) strongly suppresses the multiple hair phenotype of overexpressed *kermit*. In contrast, reduction of dynein or kinesin levels by removing one gene copy of the dynein heavy chain (*Dhc64C*, C) or kinesin heavy chain (Khc, D) enhances the *UAS-kermit* phenotypes, as does RNAi-mediated downregulation of *Khc* (E). The panels (A-E) represent high-magnification images of the dorsal wing sheet within the region framed in magenta in Figure 1A. (F) Quantification of the effects of panels (A-E), presented as on Figure 1E.

Kermit/GIPC1 physically and genetically interacts with Myosin VI (MyoVI) in mice and flies [29,40,41]. MyoVI is an actin filament-based motor protein implicated in the removal of endocytic vesicles away from the cell’s periphery [40,41]. We confirm the previously reported [29] genetic interaction between *kermit* and *jaguar* (*jar*, the *Drosophila* MyoVI homolog) demonstrating a strong suppression of the *UAS-kermit* phenotype by removal of one gene copy of *jar* (Figure 3A, F). Remarkably, downregulation of *jar* by RNAi led to a complete rescue of the *UAS-kermit* phenotype (Figure 3B, F). It thus appears probable that Kermit-mediated transport of Vang by MyoVI along actin cables, similarly to as it has been observed in mammalian inner ear [40], mislocalizes Vang away from its normal position, leading to the dominant PCP phenotypes.

A microtubule meshwork, polarized along the proximo-distal axis, exists in the apical region of wing epithelial cells and is implicated in vesicular transport of PCP components such as Fz and Flamingo [11]. Microtubule-based motor proteins dynein (minus end-directed) and kinesin (plus end-directed) are implicated in relocalization of PCP components [11,14]. We hypothesized that Kermit might redirect Vang transport from the microtubule-mediated, occurring within the apical plane of the cell and required for the proper PCP establishment, to the actin-mediated, directing Vang away from the apical membrane. If so, downregulation of the microtubule-based motor proteins would be expected to enhance the phenotypes of *kermit* overexpression. This is what we observed: removal of one gene copy of the dynein heavy chain (*Dhc64C*) or kinesin heavy chain (Khc), as well as RNAi-mediated downregulation of *Khc* led to a significant increase in the multiple wing hair phenotype induced by *UAS-kermit* (Figure 3C-F; a similar experiment with *Dhc64C-RNAi* was impossible due to lethality of wing expression of this construct). The enhancement of the *UAS-kermit* phenotype appears stronger upon reduction in dynein (Figure 3C, F); it was also seen at the level of the number of hairs produced by cell: 2.5±0.1 (vs. 2.2±0.1 in *UAS-kermit* alone; P value <0.005 form the Student’s t-test).

Thus, Kermit may differently mediate transport of Vang along actin and microtubule cables.

## Discussion

At the top of the signaling hierarchy in PCP lies a G protein-coupled receptor Fz [2,42]. The heterotrimeric Go protein emerged as an immediate transducer of Fz in *Drosophila* as well as other organisms [6,14,43]. One of the mediators of Go signaling in PCP is the endocytic GTPase Rab5 required for the proper Fz internalization and relocalization [9]. During PCP establishment, Fz concentrates at the distal apical position of wing epithelia [12]. Here we describe identification of Kermit as another transducer of Go in PCP. We find that *kermit* downregulation suppresses the *Gαo*-overexpression phenotypes, and that *Gαo* and *kermit* co-overexpression results in a prominent synergism in PCP malformations.

Kermit and its mammalian homolog GIPC, through their PDZ domain, are known to interact with a number of proteins in various organisms. We were initially tempted by the observations in *Xenopus* and mice that Kermit/GIPC could interact with members of the Fz and RGS protein families – Fz3, Fz7, and RGS19 [27,28]. Since Go also binds Fz and RGS proteins [15,16,30], we hypothesized that a quaternary complex consisting of Fz, Go, Kermit, and RGS19 could form in *Drosophila* PCP, with Kermit as a potential organizer of these interactions. However, we find that *Drosophila* Kermit does not interact with Fz. Similarly, no binding between Kermit and the *Drosophila* RGS19 homolog could be seen. Thus Kermit is unlikely to act as a scaffold in Fz-Go signaling, and another mode of action of Kermit in transducing Go signal exists in PCP.

In a recent study using mouse genetics and cellular assays, a role of GIPC1 in regulating Vangl2 (a murine homolog of *Drosophila* Vang) intracellular trafficking has been revealed [40]. In *Drosophila* PCP, Vang relocalizes to the site opposite to Fz at the proximal apical tip of wing epithelia [13]. We provide genetic evidence placing Vang downstream from Kermit in *Drosophila* PCP, suggesting that the Kermit-Vang connection is conserved from insects to mammals.

*kermit* expression is strongly upregulated in the developing wing during PCP establishment [34], and *kermit* overexpression induces strong PCP phenotypes ( [29] and this work). In *Xenopus*, both up- and down-regulation of *kermit* lead to defective Fz3-dependent neural crest induction [28]. It is thus surprising that *Drosophila kermit* loss-of-function alleles were homozygous viable and did not reveal PCP phenotypes [29]. We propose that Kermit may regulate *Drosophila* PCP redundantly with some other PDZ domain-containing proteins, such as Scribble or Patj, which genetically interact with PCP components but on their own also produce only mild phenotypes [44,45]; of those Scribble has been shown to interact with Vang both in *Drosophila* and mammals [4,44]. In general, up to 75% of genes *Drosophila* are estimated to be phenotypically silent in loss-of-function due to redundancy [46], and the significance of gain-of-function analysis in discovery of novel important pathway components has been highlighted in a recent large-scale *Drosophila*-based assay [47]. We thus consider Kermit, based on the presented overexpression and genetic interaction studies, as an important regulator of *Drosophila* PCP.

A genetic and physical interaction between Kermit and the unconventional actin-based motor MyoVI has been described [29,40,41]. We confirmed that the dominant *UAS-kermit* PCP phenotypes critically depend on the MyoVI activity. MyoVI has been previously shown to mediate removal of endocytic vesicles away from the cell’s periphery [40,41]. The excessive activity of Kermit or MyoVI may thus result in removal of Vang-containing vesicles from the apical membrane, contributing to mislocalization of Vang and appearance of the PCP defects. In contrast, microtubule-based transport along the apical microtubule cables, polarized below the apical plasma membrane in wing epithelia, mediates the correct relocalizations of Fz and Vang in PCP [11]. It is probable that a competition between the actin-based and microtubule-based motors may exist for the endocytic vesicles containing PCP components, and that excessive Kermit activity unbalances this competition in favor of the actin-based transport. We thus tested whether reduction in the levels of the microtubule-based transport system would further aggravate the dominant *UAS-kermit* PCP phenotypes. And indeed, reduction in either the minus end-directed motor dynein or the plus end-directed motor kinesin significantly enhances the *UAS-kermit* effects.

**Figure 4:**
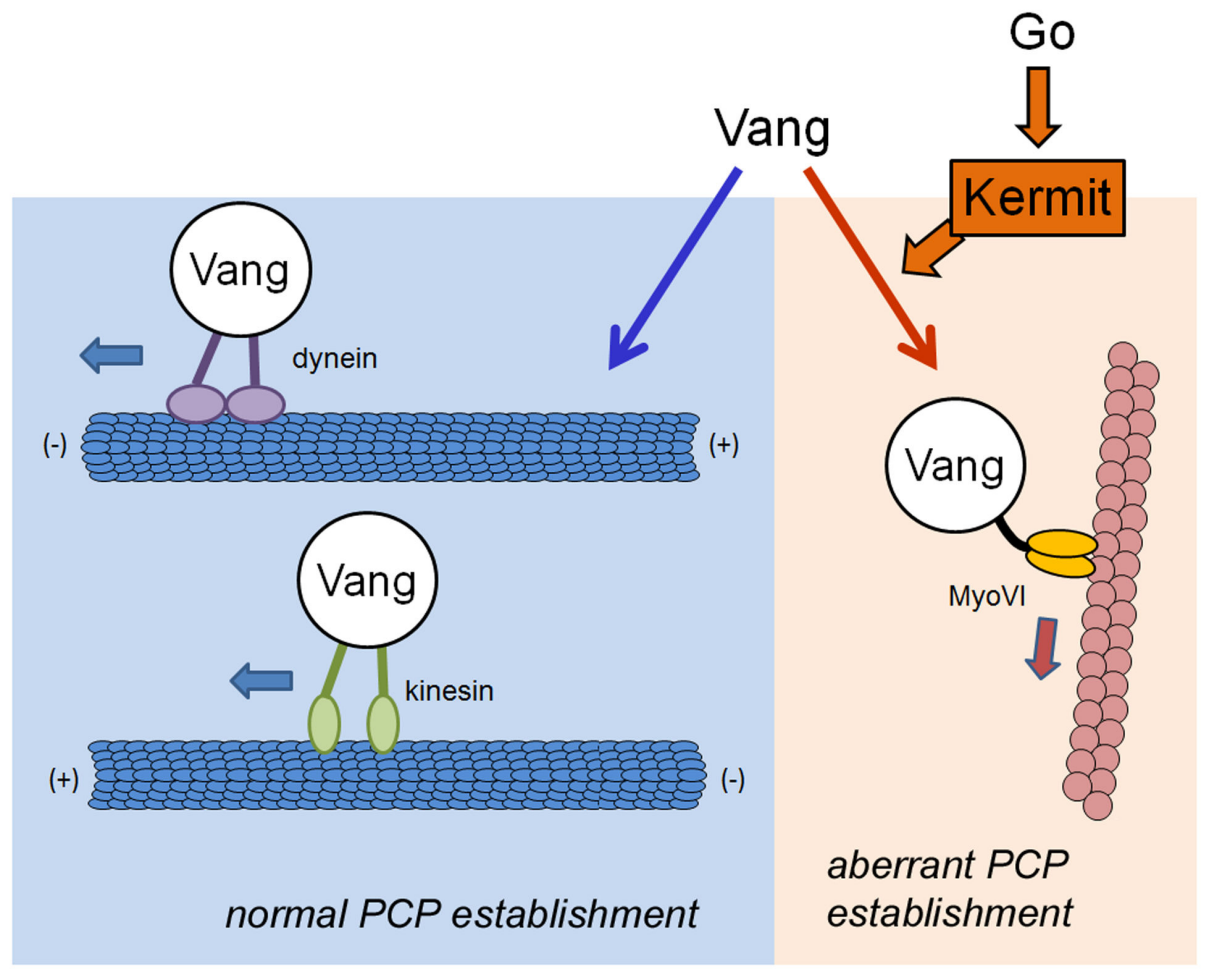
The model of interrelationship of Go, Kermit, Vang, and motor proteins in PCP. Microtubule-base motor proteins dynein and kinesin contribute to the asymmetric distribution of Vang in the apical plane, relocalizing Vang vesicles required for the PCP establishment. In contrast, the actin-based MyoVI motor contributes to remove Vang away from the apical membrane and active PCP pool. Kermit transduces the signaling from Go to promote trafficking of Vang via MyoVI.

We thus propose the following model to collectively explain our results. We speculate that endocytic vesicles containing PCP components can be transported in a planar manner, along the microtubule meshwork underlying the apical plasma membrane – the mode of transport required for the proper apical relocalizations of these components. Alternatively, the vesicles can be trapped by the actin cables and transported away from the apical membrane, removing them from the active pool of PCP components (Figure 4). In the case of Vang, the choice between these decisions is regulated by the Kermit protein, which favors the actin-based transport (Figure 4).

The activity of Kermit is controlled by Go, which in turn acts downstream from Fz. Thus, Go seems to regulate endocytosis and endocytic transport of the two key transmembrane components of PCP – Fz (through Rab5 [9]) and Vang (through Kermit [this work]).

Our findings and model shed new light on the mechanisms of complex inter-regulations ensuring the robust epithelial polarization, likely conserved across the metazoans.

## Materials and Methods

### Fly stocks

*yw, MS1096-Gal4*, *GMR-Gal4*, jar*322*/*TM3* P{Ubx-lacZ.w ^*+*^}TM3 Sb ^1^, Vang^*stbm-6*^ *,* b^*1*^ pr ^*1*^ Khc *^8^/CyO*, and Dhc64C^*4-19*^ P{FRT(w ^*hs*^)}*2A/TM6B* Tb ^1^ were from Bloomington *Drosophila* Stock Center. *UAS-RNAi-kermit* (transformant Id #109297), *UAS-RNAi-Gαo* (#19124), *UAS-RNAi-jar* (#37535), *UAS-RNAi-Vang* (#7376), *UAS-RNAi-Khc* (#44338), and *UAS-RNAi-Dhc64C* (#28054) were from Vienna *Drosophila* RNAi Center. *fz[-/-*] animals were the *fz*[*H51*]/[*P21*] transheterozygotes [37]. The kermit alleles were *kermit^SH0225^* [32] and *kermit^ex31^* [29]. To screen for *Gαo*-interacting mutations, the *UAS-Gαo* transgene on the first chromosome [6] was recombined with *MS1096-Gal4*. Twenty to thirty *MS1096-Gal4, UAS-Gαo/X; kermit*[*SH0225*]*/+* progeny flies were analyzed in two independent crosses. All crosses were performed at 25°C. Cells producing multiple hairs were counted in the dorsal region framed by the veins 3 and 4 and the intervein 1. To monitor Vang localization in pupal wings 30h after puparium formation, *Act-Stbm-YFP* flies were used as described [13].

### Gene cloning and protein expression

*kermit* cDNA (clone LP09416) was obtained from *Drosophila* Genomics Resource Center (DGRC) and subcloned into pMAL-c2X (NEB) by EcoRI and SalI after amplification using the oligonucleotides: forward: CAATCCGAATTCATCATGCCGCTCTTCAC, reverse: GGTATGGTCGACCAATTACTTGGGACTGG. Recombinant Kermit was purified according to the manufacturer’s instruction, along with MBP expressed by the parental pMAL-c2X plasmid.

For fly transformation, *kermit* was subcloned into pUAST-attb [48] by EcoRI and XhoI. Forward primer was the same as above, the reverse primer was: GGTATGCTCGAGCAATTACTTGGGACTGG. The *φχ-22A* line [48] was used for germ-line transformation to produce the *UAS-kermit* transformant on the second chromosome.

CG5036 cDNA (clone LD40005) was obtained from DGRC and subcloned into pQE-30 plasmid (Qiagen) by KpnI and SalI, primers used were: forward: GCAGGTGGTACCATGTCCTGCACCGTTTCCG, reverse: GGACATGTCGACCTAAGTTGGACTATCCG. Human RGS19 was obtained from ImaGenes and subsequently cloned into pQE-30, using the oligonucleotides: forward:

CTCCGCGGTACCATGCCCACCCCGCATG, reverse

GCTGGGGTCGACCTAGGCCTCGGAGGAG. Both plasmids along with pQE32-Gαo were used to express recombinant proteins purified as described [20]. MBP-Fz, His_6_-Rab5, and GST were produced as described previously [9].

### In Vitro Binding Assay and Western Blotting

The following proteins were covalently linked on CNBr-activated Sepharose (GE Healthcare) according to manufacturer’s instructions: His_6_-Gαo, GST, MBP, MBP-Kermit, His_6_-CG5036, and His_6_-Rab5 (Figure 1). Immobilized His_6_-Gαo was preloaded with 1mM GDP or GTPγS in the HKB buffer (50mM HEPES-NaOH, 100mM KCl, 10mM NaCl, 1mM DTT, 5mM MgCl_2_, pH7.5) for 30min at RT. Soluble proteins were added in equimolar amounts (Figure 1D, E). *Drosophila* head extracts from *GMR-Gal4; UAS-Gαo* flies were prepared as described [20]. Bacterial membranes expressing MBP-Fz were solubilized for 30min with 10mM CHAPS at 4°C prior to the pull-down protocols as described [9,15]. After incubation for 3h at 4°C, matrices were washed 5× with HKB buffer before elution with 8M Urea. The samples were resolved on 12% SDS-PAGE gel followed by Western blotting with rabbit anti-MBP (NEB, 1:4000), mouse anti-His_6_ (Qiagen, 1:2500), or rabbit anti-Gαo/i3 (Merck, 1:1000) antibodies.
